# Brief history of agricultural systems modeling

**DOI:** 10.1016/j.agsy.2016.05.014

**Published:** 2017-07

**Authors:** James W. Jones, John M. Antle, Bruno Basso, Kenneth J. Boote, Richard T. Conant, Ian Foster, H. Charles J. Godfray, Mario Herrero, Richard E. Howitt, Sander Janssen, Brian A. Keating, Rafael Munoz-Carpena, Cheryl H. Porter, Cynthia Rosenzweig, Tim R. Wheeler

**Affiliations:** a University of Florida, Agricultural and Biological Engineering Department, Museum Road, Gainesville, FL 32611, USA; b Oregon State University, USA; c Michigan State University, USA; d Colorado State University, USA; e University of Chicago, USA; f Oxford Martin Programme on the Future of Food, University of Oxford, Department of Zoology, South Parks Rd., Oxford OX1 3PS, UK; g CSIRO, Australia; h University of California-Davis, USA; i Wageningen University, Netherlands; j NASA, Columbia University, USA; k University of Reading, UK

**Keywords:** Agricultural systems, Models, Next generation, Data, History

## Abstract

Agricultural systems science generates knowledge that allows researchers to consider complex problems or take informed agricultural decisions. The rich history of this science exemplifies the diversity of systems and scales over which they operate and have been studied. Modeling, an essential tool in agricultural systems science, has been accomplished by scientists from a wide range of disciplines, who have contributed concepts and tools over more than six decades. As agricultural scientists now consider the “next generation” models, data, and knowledge products needed to meet the increasingly complex systems problems faced by society, it is important to take stock of this history and its lessons to ensure that we avoid re-invention and strive to consider all dimensions of associated challenges. To this end, we summarize here the history of agricultural systems modeling and identify lessons learned that can help guide the design and development of next generation of agricultural system tools and methods. A number of past events combined with overall technological progress in other fields have strongly contributed to the evolution of agricultural system modeling, including development of process-based bio-physical models of crops and livestock, statistical models based on historical observations, and economic optimization and simulation models at household and regional to global scales. Characteristics of agricultural systems models have varied widely depending on the systems involved, their scales, and the wide range of purposes that motivated their development and use by researchers in different disciplines. Recent trends in broader collaboration across institutions, across disciplines, and between the public and private sectors suggest that the stage is set for the major advances in agricultural systems science that are needed for the next generation of models, databases, knowledge products and decision support systems. The lessons from history should be considered to help avoid roadblocks and pitfalls as the community develops this next generation of agricultural systems models.

## Highlights

• Advances were fastest after events that caused economic or environmental concerns
• Technological advances have had major benefits on agricultural system modeling
• Progress toward robust models has been enabled through open, harmonized data
• Modularity and interoperability are features needed for next generation models
• More integration among disciplines and data are needed to advance agricultural models

## 1 Introduction

The world has become more complex in recent years due to many factors, including our growing population and its demands for more food, water, and energy, the limited arable land for expanding food production, and increasing pressures on natural resources. These factors are further compounded by climate change that will lead to many changes in the world as we have known it (e.g., Wheeler and von Braun, 2013). How can science help address these complexities? On the one hand, there is a continuing explosion in the amount of published information and data contributions from every field of science. On the other hand, the problem of managing all of this knowledge and underpinning data becomes more difficult and risks information overload. The information explosion is leading to greater recognition of the interconnectedness of what may have been treated earlier as independent components and processes. We now know that interactions among components can have major influences on responses of systems, hence it is not necessarily sufficient to draw conclusions about an overall system by studying components in isolation (Hieronymi, 2013). These interactions transcend traditional disciplinary boundaries. Although there continues to be a strong emphasis on disciplinary science that leads to greater understanding of components and individual processes, there is also an increasing emphasis on systems science.

Systems science is the study of real world “systems” that consist of components defined by specialists. These components interact with one another and with their environment to determine overall system behavior (e.g., see Wallach et al., 2014). These interacting components are exposed to an external environment that may influence the behavior of system components but the environment itself may not be affected by the changes that take place within the system boundary. Although systems are abstractions of the real world defined for specific purposes, they are highly useful in science and engineering across all fields, including agriculture. An agricultural system, or agro-ecosystem, is a collection of components that has as its overall purpose the production of crops and raising livestock to produce food, fiber, and energy from the Earth's natural resources. Such systems may also cause undesired effects on the environment.

Agricultural systems science is an interdisciplinary field that studies the behavior of complex agricultural systems. Although it is useful to study agricultural systems in nature using data collected that characterize how a particular system behaves under specific circumstances, it is impossible or impractical to do this in many situations. Scientific study of an agro-ecosystem requires a system model of components and their interactions considering agricultural production, natural resources, and human factors. Thus, models are necessary for understanding and predicting overall agro-ecosystem performance, for specific purposes. Data are needed to develop, evaluate, and run models so that when a system is studied, inferences about the real system can be simulated by conducting model-based “experiments.” When we consider the “state of agricultural systems science,” it is thus important to consider the state of agricultural system models, the data needed to develop and use them, and all of the supporting tools and information used to interpret and communicate results of agricultural systems analyses for guiding decisions and policies.

Agricultural system models play increasingly important roles in the development of sustainable land management across diverse agro-ecological and socioeconomic conditions because field and farm experiments require large amounts of resources and may still not provide sufficient information in space and time to identify appropriate and effective management practices (e.g., Teng and Penning de Vries, 1992). Models can help identify management options for maximizing sustainability goals to land managers and policymakers across space and time as long as the needed soil, management, climate, and socioeconomic information are available. They can help screen for potential risk areas where more detailed field studies can be carried out. Decision Support Systems (DSSs) are computer software programs that make use of models and other information to make site-specific recommendations for pest management (Michalski et al., 1985, Beck et al., 1989), farm financial planning (Boggess and Moss, 1989, Herrero et al., 1999), management of livestock enterprises (e.g., Herrero et al., 1998, Stuth and Stafford-Smith, 1993), and general crop and land management (Plant, 1989, Basso et al., 2013). DSS software packages have mainly been used by farm advisors and other specialists who work with farmers and policymakers (e.g. Nelson et al., 2002, Fraisse et al., 2015), although some may be used directly by farmers. In addition to this type of farm-level decision making support, agricultural system models are increasingly being used for various types of landscape-scale, national and global modeling and analysis that provides information to the general public, to inform research and development investment decisions, and informs specific public policy design and implementation.

In this paper, we provide a critical overview of past agricultural systems modeling followed by a discussion of the characteristics of this history relative to lessons learned that may help guide future progress. We discuss the state of agricultural system science relative to current and future needs for models, methods and data that are required across a range of public and private stakeholders. We start with an overview of major events that happened during the last 50 + years that led to an increased emphasis on agricultural systems modeling. This timeline identifies key drivers that led to the increasing interest and investments in agricultural system models, demonstrates the complexities of many of the issues, and illustrates a range of purposes. This is followed by an overview of the characteristics of agricultural systems models and the wide range of purposes that various researchers in different disciplines have had when developing and using them. We also discuss the key messages from this history that should help guide efforts to develop the next generation of models, databases, and knowledge-based tools.

## 2 A brief history

**Fig. 1:**
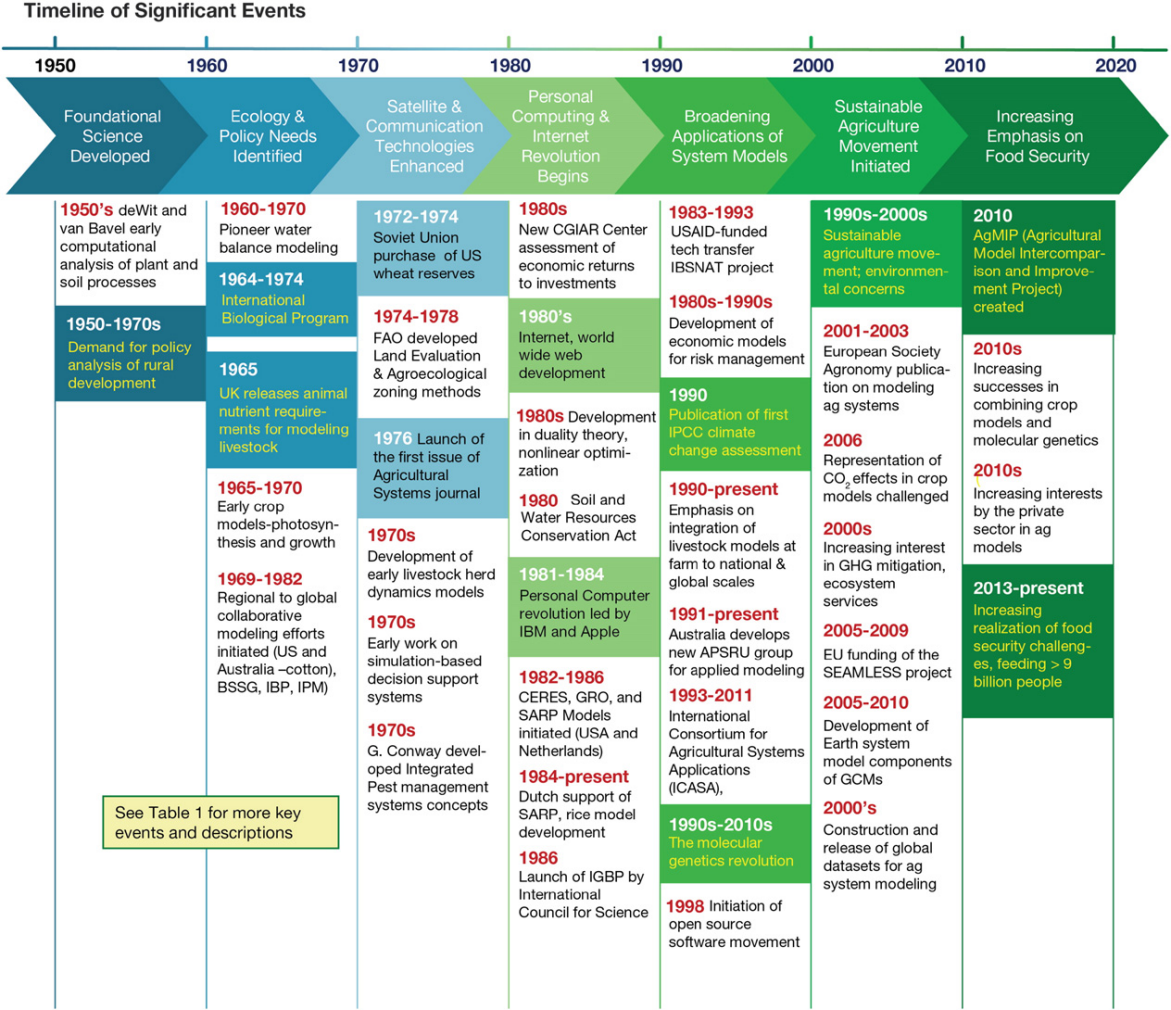
Summary timeline of selected key events and drivers that influenced the development of agricultural system models. Additional details and key events are provided in Table 1 and in the text.

The history of agricultural system modeling is characterized by a number of key events and drivers that led scientists from different disciplines to develop and use models for different purposes (Fig. 1). Some of the earliest agricultural systems modeling (Table 1) were done by Earl Heady and his students to optimize decisions at a farm scale and evaluate the effects of policies on the economic benefits of rural development (Heady, 1957, Heady and Dillon, 1964). This early work during the 1950s through the 1970s inspired additional economic modeling. Dent and Blackie (1979) included models of farming systems with economic and biological components; their book provided an important source for different disciplines to learn about agricultural systems modeling. Soon after agricultural economists started modeling farm systems, the International Biological Program (IBP) was created. This led to the development of various ecological models, including models of grasslands during the late 1960s and early 1970s, which were also used for studying grazing by livestock. The IBP was inspired by forward-looking ecological scientists to create research tools that would allow them to study the complex behavior of ecosystems as affected by a range of environmental drivers (Worthington, 1975, Van Dyne and Anway, 1976).

**Table 1:** Timeline of key events that shaped the development and use of agricultural system models.

Year	Event	Impacts
1940s–1950s	de Wit (1958) and van Bavel (1953) develop early computational analyses of plant and soil processes; Development of nutritional requirement tables for cattle (NRC, 1945)	Foundation established for the application of simulation and operations research optimization in plant-soil systems research and for modeling farm animal responses to nutrients
1950–1970s	Demand for policy analysis of rural development	Representative farm optimization models were developed and applied by Heady and students at Iowa State University, thus establishing use of linear programming methods for agricultural production
1960–1970	Pioneers in soil water balance modeling (WATBAL) [(Slatyer, 1960, Slatyer, 1964, Keig and McAlpine, 1969; Ritchie, 1972; McCown, 1973)]	Water balance models proved to be useful in the evaluation of climatic constraints to agricultural development. Foundations for linking soil and plant models established.
1964–1974	International Biological Program	Strong emphasis on large scale ecological and environmental studies led to development of grassland ecosystem models; laid foundation for ongoing work today
1965	UK releases nutrient requirement tables for ruminants (ARC, 1965, first work since the 50s)	Very influential publication; subsequent development of feeding systems models throughout Europe.
1965–70	Early crop modeling pioneers develop photosynthesis and growth models (C. T. de Wit, W. G. Duncan, R. Loomis)	Captured imagination of many crop and soil scientists. Prompted many to follow in their steps.
1969–75	S-69 Cotton Systems Analysis Project (Bowen et al., 1973, Stapleton et al., 1973, Jones et al., 1974, Jones et al., 1980, Baker et al., 1983)	Prompted development of several cotton models (W. G. Duncan, J. D. Hesketh, D. Baker, J. Jones, J. McKinion)
1971	Creation of the Biological System Simulation Group (BSSG)	Led to self-supported annual workshops aimed at advancing cropping system and other biological system models, continuing through 2014
1970s and early 80s	Development of early herd dynamics simulation models (Freer et al., 1970, IADB, 1975, Davis et al., 1976, ILCA, 1978, Sanders and Cartwright, 1979, Konandreas and Anderson, 1982)	Established in the developed world but some early examples in the developing world. Crucial for the advancement of whole livestock farm modeling and for representing disease and reproductive impacts
1970s	Gordon Conway develops concept of IPM in Malaysia. Huffaker Integrated Pest Management (IPM) Project begins in USA, evolves into the Consortium for IPM, ending in 1985. Global emphasis on reducing pesticide use, due to major increases in pesticide use globally and resistance in target pest populations.	Insect and disease models developed and used to help establish economic thresholds and to predict timing of threshold exceedance; some pest models were linked with crop models
Mid 1970s	Discovery of chaos in ecological systems by Robert May (May, 1976) and related advances in theoretical population ecology	Led to new approaches to modeling predator-prey, host-disease interactions
1972–74	Soviet Union purchase of US wheat reserves, causing major price spike (see Pinter et al., 2003)	US Government created LACIE, AGRISTARS projects to develop and use crop models with remote sensing to obtain strategic crop forecasts. Led to development of CERES-Wheat and CERES-Maize models (first published in 1986)
1974–1978	FAO development of Land Evaluation Framework in 1974 and an automated Agro-Ecological Zoning (AEZ) in 1978. (FAO, 1976, FAO, 1978–81)	Provided first methodology for land evaluation on a global basis, integrating soil, climate, vegetation, and socio-economic factors, leading to many applications and efforts to improve integrated assessment approaches
1975–1982	Early pioneers in computer simulation based decision support — SIROTAC and Australian Cotton Industry (CSIRO, 1980); S-107 Project on soybean modeling in the US	The Australian cotton modeling was the first major initiative to put crop and pest models in the hands of farmers for decision support. The soybean project in the US led to development of two major soybean models SOYGRO (Wilkerson et al., 1983) and GLYCIM (Acock et al.,1985).
1976	Launch of the first issue of Agricultural Systems, edited by C. R. W. Spedding (Spedding, 1976)	This journal helped legitimize agricultural system modeling, providing a place for scientists to publish their agricultural systems modeling and analyses as well as a collection of scholarly work in this area. This journal continues today with impact factor of about 2.5
1979	E.R. Orskov establishes the ‘Dacron bag technique’ for measuring the degradability of feed in the rumen (Orskov and McDonald, 1979)	Very influential method developed for characterizing the nutritional value of feeds, opening possibilities of new types of models; a new era of dynamic feed characterization started, leading to better animal models
1980	Soil and Water Resources Conservation Act analysis for 1980, mandate to develop a model to predict impacts of soil erosion on crop productivity	The comprehensive soil-cropping system model, (EPIC, the Environmental Policy Integrated Climate model), was developed to estimate soil productivity as affected by erosion
1980s	Growth of CGIAR Centers creates demand for assessment of economic returns to investments in agricultural research	Market surplus methods developed for estimating economic returns to investments, demonstrating high returns to agriculture research investment
1981–1984	Personal computer (PC) revolution led by IBM introduction of its Model 5150 personal computer and the first Apple Mac computer in 1984	These new PCs led to major increases in individual access to computer power; many agricultural models began appearing on PCs
1981	Development of the first soil nitrogen (N) model for predicting crop responses under both water and N limiting conditions (Seligman and van Keulen, 1981)	This model was the foundation for future soil N models in APSIM, DSSAT, and other suites of crop models
1980s through early 1990s	Development and growth of the Internet that began to connect computers globally	Ushered in new era of global communication and information technologies that has affected all areas of our lives, including agricultural system model development and use
1982 to 1986	CERES Models (Maize and Wheat) and GRO (SOYGRO and PNUTGRO) models were developed (Wilkerson et al., 1983, Boote et al., 1986)	The CERES models linked soil water, soil nitrogen and crop growth and yield together in a comprehensive fashion for the first time. They stimulated interest and activity in crop modeling in many parts of the world.
1980s	Development of duality theory and advances in nonlinear optimization via development of GAMS by World Bank	Led to advances in applications of econometric methods for production model estimation and to national and regional policy analysis models; use of new entropy methods reduced data requirements for the models
1980–1990	Influential developments in pasture modeling (Hurley pasture model — Johnson and Thornley, 1983 and the SAVANNA model (Coughenour et al., 1984)	Led to a proliferation of pasture models for intensive temperate and tropical grasslands and savanna systems. These models simulated herbage mass and accounted for sward components, which led to a more sophisticated representation of grazing processes.
1983–1993; DSSAT continuing today	USAID funded international IBSNAT project for facilitating technology transfer using systems approaches and crop and soil models	This led to the creation of the DSSAT suite of crop models that combined the CERES family of models with the SOYGRO and PNUTGRO models. The availability of the IBSNAT guidelines for data collection for crop modeling strengthened the crop model testing effort around the world.
1984 –continuing today	Dutch Government funding of the SARP (Systems Analysis of Rice Production) project at IRRI in the Philippines.	Development of a dynamic rice model that later was named ORYZA, which is still widely used today (Penning de Vries et al., 1991)
1985–1992	Earliest application of crop-soil systems models in a developing country “research for development” context — Kenya-Australia Dryland Farming Systems Project (McCown et al., 1992, Keating et al., 1991)	First PC used in agricultural research in Kenya running CERES Maize (influenced strongly by the IBSNAT minimum data set guidance) in 1985. Formed the foundation for modeling low input subsistence agricultural systems and exploring development opportunities. This experience went on to strongly influence the evolution of the APSIM farming systems simulator.
1986	Launch of the IGBP (International Geosphere-Biosphere Program) by the International Council for Science (ICSU)	Brought attention to the planet under pressure, including climate change, and helped coordinate research at regional and global scales on interactions of Earth's biological, chemical, physical, and human systems, including influence on ecosystem modeling
1970s–1980s	Development of optimization and econometric methods for application to production risks	Broadened analysis of production to include risk management behavior (see Anderson et al., 1977, Just and Pope, 1978, Antle, 1983, Antle, 1987)
1980s until now	Modeling herd replacement decisions with dynamic programming (Van Arendonk and Dijkhuizen, 1985)	As computer power increased, more complex applications attempting to optimize intensive and industrial livestock production occurred.
1990	Publication of the first Intergovernmental Panel on Climate Change (IPCC, 1990) Assessment Report	Led to first use of crop and economic models for climate change impact assessments on crops at field to global-scales (e.g., Curry et al., 1990, Rosenzweig and Parry, 1994); led to broad use of agricultural and ecological models that estimate GHG emissions and carbon dynamics and economic models for assessing impacts of climate change on agriculture
1990s until now	The era of livestock systems model integration (Herrero et al., 1996, Herrero et al., 1999, Freer and Donnelly, 1997)	Many soft ‘modular’ couplings of simulation models of individual animal performance, herd dynamics, pasture and crop models happened at this time.
1990–1994	First studies on global impacts of potential climate change on agricultural systems (Rosenzweig and Parry, 1994)	These were the first studies making broad use of crop and economic models for global impacts. These studies paved the way for many other national and global impact studies of climate change impacts and adaptation.
1991–continuing today	Australian governments develop a new APSRU group for modeling agricultural systems for practical uses	This led to the now widely used APSIM (McCown et al., 1996, Keating et al., 1991, Keating et al., 2003) suite of cropping system models which drew on early experience with CERES, EPIC and PERFECT models but re-engineered the “farming systems” foundations.
1992	Comprehensive, model-based scenario analysis funded by the European Union for policy decisions	Grounds for Choice published (Netherlands Scientific Council for Government Policy, 1992). Grounds for Choice.
1992	The Cornell Net Carbohydrate and Protein System is launched (Russell et al., 1992)	The CNCPS became the first commercially available dynamic model of digestion in ruminants. Its development influenced the current livestock performance models in many parts of the world.
1993–2011	International Consortium for Agricultural Systems Applications (ICASA), formed in 1993, ended in 2011	Helped crop modelers collaborate to develop standards for input data for crop models (Hunt et al., 1994), leading later to the ICASA data dictionary and data standards (White et al., 2013), now used in harmonizing model inputs in AgMIP project (White et al., 2013).
1998	Initiation of open source software movement, leading to more collaborative software development	Led to interest in providing open-source versions of widely-used crop simulation models; now being done by some ag system modelers (e.g., APSIM, DSSAT).
1999	The Livestock Revolution study (Delgado et al., 1999)	Key analysis explaining projected growth of livestock sector showing that ‘as people get richer and societies urbanize they consume more livestock’. Led to acknowledgement of need for increased understanding of livestock sector for agricultural development.
1980s–1990s	Interest in trade liberalization	Led to quantitative analysis of trade policies and development of national and global agricultural trade policy models.
1990s–2010s	The molecular genetics revolution: Genome sequencing technological advances and advances in understanding of the functions of crop and animal genes; ability to genotype new lines and breeds	Led to still evolving efforts by various public crop modeling groups and by seed companies to connect ecophysiological crop models for plant breeding and management purposes (e.g., see White and Hoogenboom, 1996, Hoogenboom and White, 2003, Hammer et al., 2006, Messina et al., 2006).
1990s–2000s	Sustainable agriculture movement; greater concern on environmental consequences of agriculture	Led to incorporation of biophysical processes into farm household, econometric and programming approaches; also led to development of “tradeoff analysis” approach; spatial data and tools increasingly used to develop spatially explicit biophysical and economic models
Late 1990s–2000s	Construction and release of global datasets of cropping areas, sowing dates and yields (Ramankutty and Foley, 1999, Ramankutty et al., 2008)	Allowed researchers to run simulations at finer resolution over greater model domains with more clearly documented assumptions and inputs.
2000s	Increasing interest in greenhouse gas (GHG) mitigation and the importance of ecosystem services	Led to models for analysis of mitigation of GHG in agriculture via soil C sequestration, afforestation, reduced livestock emissions; also led to linkages of economic models with crop, livestock, hydrology, and ecosystem models.
2001–2003	European Society Agronomy meeting hosts special session on modeling cropping systems. Published as Volume 18 European Journal Agronomy	This meeting led to a special issue of European Journal of Agronomy (vol 18) in which comprehensive papers on the major modeling systems, namely DSSAT, APSIM, CROPSYST, STICS, Wageningen models. Over 2000 citations for models in this publication.
2006	Representation of CO_2_ effects in crop model simulations challenged by Long et al. (2006)	Opened a debate between plant experimenters and modelers on the skill of crop models for yield prediction in future climates; prompted interest in more evaluations of CO_2_ effects interacting with temperature, other factors
2005–2009	European Union funding of the System for Environmental and Agricultural Modeling: Linking European Science and Society (SEAMLESS)	This led to major collaboration across Europe for developing models for use across scales, from field to farm, country, and EU levels.
2005–2010	Development of Earth system models, components of general circulation models (GCMs)	Led to new methods for coupling crop simulation models to land surface schemes of numerical climate models (Challinor et al., 2004).
2006	FAO Livestock's Long Shadow report (Steinfeld et al., 2006)	Demonstrated the large environmental footprint of livestock leading to programs for assessing and reducing the environmental impacts of livestock. Most of this work was done through modeling.
Mid 2005s onwards	Development of global livestock models (Bouwman et al., 2005, FAO, 2013, Herrero et al., 2013)	Global integrated assessment of livestock systems now possible at high resolution including land use, emissions, economics, biomass use and others (Havlik et al., 2014, Cohn et al., 2014, PBL Netherlands Environmental Assessment Agency, 2013, Bouwman et al., 2013 and others) and their links to other sectors (crops, forestry, energy, etc.).
2010	Creation of the Agricultural Model Intercomparison and Improvement Project (AgMIP), a global program and community of agricultural scientists	This initiative led to model comparisons and initiatives for improving models, capturing the imagination and interest of agricultural modelers worldwide (Rosenzweig et al., 2013a, Rosenzweig et al., 2013b, Asseng et al., 2013).
2010s	Increasing interests by the private sector in agricultural system models	Some companies create their own crop modeling teams, others start working in public-private collaborations.
2010s	With the food price shock of 2008/2010, a realization of the need to increase food production to meet needs of 10 billion by 2050, including challenges of climate change and sustainable natural resources	This realization is leading to greater interest in use of new ICT developments (e.g., cloud computing, smart phones, app stores, mobile computing, use of UAVs for agricultural management) and agricultural system models to help guide investments and development and to greater interest by the private sector.

The IBP initiative brought together scientists from different countries, different types of government, and different attitudes toward science (Breymeyer, 1980). Before this program, systems modeling and analysis were not practiced in scientific efforts to understand complex natural systems. IBP left a legacy of thinking and conceptual and mathematical modeling that contributed strongly to the evolution of systems approaches for studying natural systems and their interactions with other components of more comprehensive, managed systems (Coleman et al., 2004).

Models of agricultural production systems were first conceived of in the 1960s. One of the pioneers of agricultural system modeling was a physicist, C. T. de Wit of Wageningen University, who, in the mid-1960s, believed that agricultural systems could be modeled by combining physical and biological principles. Another pioneer was a chemical engineer, W. G. Duncan, who had made a fortune in the fertilizer industry and returned to graduate school to obtain his PhD degree in Agronomy at age 58. His paper on modeling canopy photosynthesis (Duncan et al., 1967) is an enduring development that has been cited and used by many crop modeling groups since its publication. After his PhD degree, he began creating some of the first crop-specific simulation models (for corn, cotton, and peanut, see Duncan, 1972). His work and the work by de Wit (1958); also see Bouman and Rabbinge (1996) intrigued many scientists and engineers who started developing and using crop models. In 1969, a regional research project was initiated in the USA to develop and use production system models for improving cotton production, building on the ideas of de Wit, Duncan, and Herb Stapleton (Stapleton et al., 1973), an agricultural engineer in Arizona. Thus, some of the first crop models were curiosity-driven with scientists and engineers from different disciplines developing new ways of studying agricultural systems that differed from traditional reductionist approaches, and inspiring others to get involved in a new, risky research approach. During this early time period, most agricultural scientists were highly skeptical of the value of quantitative, systems approaches and models.

In 1972, the development of crop models received a major boost after the US government was surprised by large purchases of wheat by the Soviet Union, causing major price increases and global wheat shortages (Pinter et al., 2003). New research programs were funded to create crop models that would allow the USA to use them with newly-available remote sensing information to predict the production of major crops that were grown anywhere in the world and traded internationally. This led to the development of the CERES-Wheat and CERES-Maize crop models by Joe Ritchie and his colleagues in Texas (Ritchie and Otter, 1984, Jones and Kiniry, 1986). These two models have continually evolved and are now contained in the DSSAT suite of crop models (Jones et al., 2003, Hoogenboom et al., 2012).

During much of the time since the 1960s, only small fractions of agricultural research funding were used to support agricultural system models, although the Dutch modeling group of C. T. de Wit was a notable exception (Bouman and Rabbinge, 1996). Thus, most of those who were modeling cropping systems, for example, struggled to obtain financial support for the experimental and modeling research needed to develop new models or to evaluate and improve existing ones. Instead, there were other “crisis” events or realizations of key needs fueling model development (Table 1), each typically leading to infusion of additional financial support over short durations of time for model development or use.

The concept of Integrated Pest Management emerged in the 1970s, in particular from the work of Gordon Conway on the pests and diseases of plantation crops in Malaysia (see Conway, 1987). In 1972, the so-called Huffaker Integrated Pest Management (IPM) project was funded in the USA to address the major problems associated with increasing pesticide use and development of resistance to pesticides by many of the target insects and diseases (Pimentel and Peshin, 2014). Mathematical models of insect pests and crop and livestock diseases had been developed starting during the first half of the 20th century, though the success of synthetic agrichemicals led to a shift in attention to other control measures in the years after the Second World War. The Huffaker project infused funds for developing insect and disease models of several crops, combined with experimental efforts aimed at reducing pesticide use and more effective use of all measures to prevent economic damage to major crops in the USA. This project continued until 1985 (as the Consortium for IPM after 1978). Coincident with this project was a major increase in the sophistication of population dynamic models in ecology and a growing appreciation of the importance of nonlinearities and the problems for forecasting they imply (May, 1976). Lively debate about the appropriate way to model ecological interactions in agricultural settings characterized these decades (e.g., see Dempster, 1983, Hassell, 1986, Gutierrez et al., 1994; and Murdoch, 1994).

Globally, the FAO and various countries were also promoting IPM, with modeling as one of the approaches used to understand how to manage pests and diseases with minimal pesticide use. During this time period, a number of insect and disease dynamic models were developed, and some were coupled with cotton and soybean crop models (Wilkerson et al., 1983, Batchelor et al., 1993), including the SOYGRO model that is now in DSSAT (Jones et al., 2003). This period of time also led to the development of a general framework for coupling crop models with insect and disease information to estimate impacts on growth and yield (Boote et al., 1983).

Due to increased emphasis on interdisciplinary research of agro-ecosystems and the need to publish scientific advances in this area, the journal Agricultural Systems was launched in 1976 (Spedding, 1976). This journal helped legitimize agricultural system modeling and provided a place for scientists to publish models and systems analyses, creating a collection of scholarly work in this area. Through its publication examples, it has continued to provide encouragement to authors across all agricultural science disciplines who have worked in agricultural systems research since 1976.

The work started by the early pioneers has continued to evolve throughout the years. Notably, Wageningen University has carried on the legacy of C. T. de Wit by training many agricultural system modelers and by developing a number of crop models that are still in use today (Penning de Vries et al., 1991, Bouman and Rabbinge, 1996, van Ittersum et al., 2003). Similarly, some of the early work of Duncan, Ritchie, and others has evolved and contributed to the widely-used DSSAT suite of crop models through collaborative efforts among the University of Hawaii, University of Florida, Michigan State University, the International Fertilizer Development Institute, Washington State University, and others (IBSNAT, 1984, Tsuji et al., 1998, Uehara and Tsuji, 1998, Jones et al., 2003, Hoogenboom et al., 2012).

There were other notable government-funded initiatives in the U.S., Netherlands, and Australia that led to major developments of crop, livestock, and economic models. This includes the 1980 US Soil and Water Conservation Act that led to development of the EPIC model that is still in use today (Williams et al., 1983, Williams et al., 1989), the USAID-funded IBSNAT project that led to the creation of the DSSAT suite of crop models that incorporated the CERES and CROPGRO models (Jones, 1993, Boote et al., 1998, Boote et al., 2010, Jones et al., 2003, Hoogenboom et al., 2012), and the Systems Analysis of Rice Production (SARP) project funded by the Dutch government starting in 1984 that led to the development of the ORYZA rice crop model, now widely used globally (Penning de Vries et al., 1991, Bouman et al., 2001). The establishment of the first fully-funded, multidisciplinary crop modeling-oriented research group in Australia in the early 1990s led to the development of the APSIM suite of cropping system models. This was a major milestone; APSIM is currently one of the most widely-used suites of models (McCown et al., 1996, Keating et al., 2003). Another major event was the development of the SEAMLESS project, funded in 2005 and operated for 5 years. This effort led to major collaboration among agricultural systems modelers and scientists across Europe for development of new data interfaces and models, and to development and integration of models at field, farm, and broader spatial scales, including cropping system and socioeconomic models (van Ittersum et al., 2008).

The evolution of economic models for different scales and purposes progressed steadily during the last five decades (Table 1). These developments were fueled by various needs at national and international levels as well as innovations in modeling approaches by the agricultural economics community. The needs included mandates of CGIAR Centers to evaluate returns on investments in research for development, the increased interest in liberalizing global agricultural trade, the evaluation of ecosystem services, and impacts of climate change and adaptation (Rosenzweig and Parry, 1994, Curry et al., 1990, Thornton et al., 2006, Nelson et al., 2009). This steady progress included the development of agricultural risk management analyses, evaluation of national, regional and global policies, and integration of other models with economic models for more holistic assessments, including crop, livestock, grassland, and hydrology models (Havlik et al., 2014, Nelson et al., 2013, Rosegrant et al., 2009, De Fraiture et al., 2007, Elliott et al., 2014a, Elliott et al., 2014b).

In parallel with these events that brought significant funding into development and use of agricultural system models, other events also contributed significantly to this evolution. The introduction of the first IBM personal computer (PC) in 1981 and the Apple Mac computer in 1984 led to widespread availability of computers during the 1980s. Afterward, individual researchers could work with agricultural system models that were being made available on personal computers or develop their own models. The PC revolution led to many innovations in other fields that have contributed to modeling of agricultural systems, such as computer graphics, statistical analysis, GIS, and other software being made available on desktops, notebooks, and smart phones. In addition, the development of the internet and world-wide web in the 1980s ushered in a new era of communication and technologies that led to greater collaboration among scientists, more rapid development of agricultural models, and improved access to data.

Another innovation in computer software development is noteworthy. In 1998, the concept of open source software was developed. As the agricultural systems science community is evolving, there is considerable interest in creating open-source agricultural system models, with modular components and with interfaces to common databases. Already, at least two cropping system models are being offered as modified open source (APSIM, https://www.apsim.info/AboutUs.aspx; and DSSAT, Cropping System Model, http://dssat.net/downloads/dssat-v46). These two crop modeling systems allow free access to model source code to enable community-based development of model components for possible inclusion in official model versions.

In parallel to funded initiatives, scientists started creating consortia and networks to enhance collaboration for specific purposes. One example was the International Consortium for Agricultural Systems Applications (ICASA; Ritchie, 1996, Bouma and Jones, 2001), which was formed in 1993 and developed data standards for use with crop models (Hunt et al., 1994, White et al., 2013). Another key development was in the construction and release of global datasets of cropping areas, sowing dates, yields, and other management inputs (Ramankutty and Foley, 1999, You et al., 2006, Monfreda et al., 2008, Ramankutty et al., 2008, Fritz et al., 2013). A milestone was reached when these global cropland cover products were developed and used for regional and global analyses of agricultural systems. Without access to data for developing, testing, and applying the agricultural system models, they are not effective.

Several events in Table 1 are associated with climate change in various ways that individually and collectively contributed strongly to advances in agricultural system models. An early contributor to modeling climate change impacts was the International Geosphere-Biosphere Program (IGBP), formed in 1986. This global project led to increasing interest in climate change and the use of models to assess what likely impacts might be under future climate conditions. Included in this work was a project on agriculture (Global Change and Terrestrial Ecosystems, or GCTE; Steffen et al., 1992). This project led to collaboration among crop modelers, who were beginning to see the need for comparing different models (e.g., Jamieson et al., 1998). An early motivation for model use in climate change research was the publication of the first IPCC assessment report on climate change (IPCC, 1990). This led to the use of crop, livestock, and economic models to assess climate change impacts on agriculture as well as agricultural adaptation and mitigation options (Rosenzweig and Parry, 1994). This then prompted crop modelers to incorporate CO_2_ effects on crop growth and yield if this effect was missing, and to use the models to perform simulation experiments using current and future projected climate conditions (e.g., Curry et al., 1990, Tubiello et al., 2002, Waha et al., 2013). These simulated changes in crop productivity were used in socioeconomic models to evaluate impacts on agricultural trade, food prices, and distribution of impacts (e.g., Rosenzweig and Parry, 1994, Adams et al., 1990, Fischer et al., 1995). Many studies have been conducted since the first work that was led by Rosenzweig, Parry, and others, in particular to provide information for subsequent IPCC assessments as well as various national and regional assessments (Fischer et al., 1995, Rosenzweig and Parry, 1994, Parry et al., 2004).

Unfortunately, these assessments used existing models, and funding did not provide support for improving and evaluating the models. Long et al. (2006) challenged the findings of crop models that had been developed using older data, particularly results suggesting that the positive fertilization effects of CO_2_ would offset the negative effects of rising temperature and lower soil moisture. Much more data are now available from FACE (Free Air CO_2_ Experiments) and T-FACE (Temperature FACE) experiments to more comprehensively evaluate and improve the interactive effects of temperature, soil moisture, and CO_2_ in current models (Kimball, 2010, Boote et al., 2010). Conducting such evaluations and improvements is one of the goals of the Agricultural Model Intercomparison and Improvement Project (AgMIP; see www.agmip.org; Rosenzweig et al., 2013a, Thorburn et al., 2014).

The creation of AgMIP in 2010 is another major milestone in the evolution of agricultural models. This initiative created a global community of agricultural system modelers with the goals of comparing and improving crop, livestock, and socioeconomic models, and using the improved models for assessing impacts and adaptation to climate change and climate variability at local to global scales, including evaluating the uncertainties of those assessments (e.g., see Asseng et al., 2013, Bassu et al., 2014, Li et al., 2015, Rosenzweig et al., 2013b). Since its start, AgMIP has promoted collaboration among virtually all agricultural modeling groups globally, creating new opportunities for substantially improving abilities to understand and predict agricultural responses to climate, including interacting effects of CO_2_, temperature, and water.

Finally, the increasing interest in improving the representation of the Earth's land area in regional and global climate models has led to new approaches for modeling agricultural systems (Osborne et al., 2009). This work has led various modeling groups to develop models that represent CO_2_, water, and GHG fluxes and also crop growth and yield of grid-cell areas (e.g., Fischer et al., 1995, Rosenzweig et al., 2013b, Elliott et al., 2014a, Elliott et al., 2014b). On the livestock side, global gridded models for feed consumption, productivity, manure production, and greenhouse gas emissions for dairy, beef, small ruminants and pork and poultry are now available (Herrero et al., 2013, FAO, 2013).

Three recent developments shown in Table 1 offer the potential for major advances in modeling agricultural systems, but these impacts have yet to be realized. The first one is the molecular genetics revolution. During the last 20 years, the progress in mapping genomes of major crops has been impressive, and the technological advances in performing DNA analyses on plants and animals have led to rapid and inexpensive genotyping that resulted in major changes in plant and livestock breeding. The potential value of this molecular genetics information includes the abilities of crop and livestock models to predict performance of crop varieties and animal breeds in specific climate and management conditions. Early work on this has shown that it is promising, yet considerably more work is needed to quantitatively link genes to physiological performance (e.g., see White and Hoogenboom, 1996, Hoogenboom and White, 2003, Messina et al., 2006, Hammer et al., 2006). The molecular biology revolution is also leading to the development of new genetic strategies for pest and disease control that are likely to be ready for regulatory study in the next decade, and this may lead to new demands for systems models to explore their efficacy and safety.

The second entry in Table 1 that holds unrealized promise is greater collaboration among public and private researchers. For example, the private sector invests heavily in data collection as part of their plant breeding process. Some companies have shown interest in providing some of those data for use in evaluating and improving models in the public sector (e.g., Gustafson et al., 2014, Kumudini et al., 2014). In addition, private companies realize that agricultural system models are valuable for evaluating new technologies and for providing benefit to their customers through better decision making. This is seen with the creation of CIMSANS (see www.ilsi.org/ResearchFoundation/CIMSANS), a public-private partnership in the International Life Sciences Institute to address sustainable agriculture and nutrition security using agricultural models. Finally, the private sector is heavily invested in molecular genetics usage in plant breeding, and some companies incorporate crop models in their plant breeding efforts (e.g., Messina et al., 2011). This provides an opportunity for public and private researchers to work together to produce more reliable models of crops and breeds for greater use of these methods in the future.

A third entry in the table that presents major opportunities for advancing agricultural system models, databases, and knowledge products is the evolution of Information and Computer Technologies (ICT). This complements the second point but is also distinct from it. In particular, ICT is likely to lead to more user-driven knowledge products and their linkage to model development. These opportunities are elaborated in the papers by Antle et al. (2017b--in this issue) and Janssen et al. (2017--in this issue).

Other events have contributed to development of specific agricultural models in different countries. We do not attempt to create a comprehensive list of all such events, but instead to highlight those that played major roles in getting this work started in addition to those that had major implications globally. Between events in Table 1, model development and use has proceeded, but overall progress has been slow at times. The continued dedication to develop reliable models has been one of the main features of many agricultural modeling efforts for cropping systems, livestock, and economics (e.g., DSSAT, EPIC, APSIM, STICS, WOFOST, ORYZA, CROPSYST, RZWQM, TOA, IMPACT, SWAP, and GTAP).

## 3 Characteristics of agricultural system models

Although many factors have motivated the development of agricultural system models, there are three characteristics that stand out among them: 1) intended use of models, 2) approaches taken to develop the models, and 3) their target scales. Here, we present these important characteristics with examples for each.

### 3.1 Purposes for model development

There are two broad categories that motivate agricultural model development; scientific understanding, and decision/policy support (e.g., Loomis et al., 1979, Passioura, 1996, Boote et al., 1996, Bouman and Rabbinge, 1996, van Ittersum et al., 2003, Ritchie, 1991, McCown et al., 1996). The first of these motivations is to increase basic scientific understanding of components of agricultural systems or understanding of interactions that lead to overall responses of those systems. Van Ittersum et al. (1998) referred to models with this purpose as explanatory. Models developed to increase scientific understanding tend to be mechanistic models as they are usually based on known or hypothesized control of physical, chemical, and biological processes occurring in crop or animal production systems. Examples are mechanistic models of photosynthesis (e.g., Farquhar et al., 1980) and water movement in soils (e.g., model implementation of the Richards (1931) equation).

At the basic science level, models developed to increase understanding are used as tools to address research questions about control of processes, magnitudes of responses, and interactions. Modeled outputs are compared with observations that are measured in laboratories or in fields for testing the understanding that is embedded in the model (Passioura, 1996). For example, transport of water or mineral N through a soil involves many processes that affect the correct balance of water or N. Likewise, the flux of carbon dioxide in a field can be measured instantaneously in flux-site experiments. There are, however, many contributors to CO_2_ flux including photosynthesis, aerial crop respiration, root respiration, and soil organism respiration, all of which are affected by the aerial and soil environment as well as by crop type, age, and condition. For livestock, the partitioning of nutrients for different physiological functions (growth, lactation, pregnancy and others) and the control of voluntary feed intake as well as their interactions and feedbacks have received considerable attention (Forbes, 1986, Illius and Allen, 1994).

Models developed to increase scientific understanding typically describe processes at fine time scales (e.g., instantaneous photosynthesis and transpiration processes, hourly nutrient supply in animals). These explanatory models of agricultural systems typically include a large number of parameters, some of which may be unknown or only known with relatively large uncertainties. And they may require other explanatory input information that may not be readily available for general applications, such as the spatial variations in the relationship between soil water and water potential. Also, uncertainties in some of the hypotheses and assumptions used in developing mechanistic models make outputs uncertain and often less useful to those outside of the modeling group.

Functional models (Addiscott and Wagenet, 1985, Ritchie and Alagarswamy, 2002), which may also be referred to as phenomenological models (developed by using data to model relationships), are based on empirical functions that approximate complex processes, such as a crop's interception of energy using plant leaf area (for estimating radiation interception or as an indicator of crop biomass) and radiation use efficiency (RUE — a measure of biomass produced per unit of radiation intercepted). This type of function requires field data to estimate RUE and usually produces reasonable results when compared to field measurements. Another example of an empirical approach is the simulation of potential evapotranspiration using the well-known functional Penman-Monteith or Priestley-Taylor equations (Allen et al., 1998), which have been used successfully for decades even though they are highly simplified compared to more mechanistic evapotranspiration models.

Explanatory models may include various combinations of mechanistic and functional model components. The choice of relationships used by different modeling groups to represent processes and components is one of the main reasons that there have been multiple models developed of the same crop, livestock, and farming systems. For these reasons, currently developed agricultural system models differ in levels of complexity, parameter and input requirements, and in their accuracy in predicting system performance. This has been demonstrated recently by the AgMIP wheat and maize model intercomparison studies that found large variations among multiple wheat and maize model yield predictions. The median of multiple models was a better predictor of crop yield across multiple sites than any single model in these studies (Asseng et al., 2013, Bassu et al., 2014).

The second overall purpose for developing models, to provide information for supporting decisions and policies, requires models that describe how the agricultural system responds to the external environmental drivers as well as decisions or policies under consideration (referred to as descriptive models by Van Ittersum et al., 1998). On the one hand, independent model-based analysis may be used to provide information useful to society for both public and private decision making. On the other hand, analyses are done to support specific public policy processes and decisions. Users of such models may be interested in prediction of responses that would help guide decisions, or they may be interested in how the system would respond if a particular decision was made. They may want to analyze alternative designs of agricultural systems or explore responses to different policies at crop, livestock, farm, or regional scales (Thornton and Herrero, 2001, Van Ittersum et al., 1998). Such models may or may not increase scientific understanding and they may have varying degrees of explanatory mechanisms; some may be purely statistical. But the key requirement is that these models provide reliable system response information that decision and policy makers need.

Models for increasing scientific understanding of agricultural systems will continue to be pursued using various scales and approaches. While these models form the basis for the decision-enabling modeling, our focus is on next generation agricultural system models for use in planning and strategic decision analyses. A key task is the evaluation of tradeoffs among possibly conflicting objectives of decision/policy makers at various levels, from field and pasture to farm, landscape, and regional scales, and for smallholder to large industrial scale farmers.

### 3.2 Approaches for modeling agricultural systems

Several dimensions are needed to describe the types of models that have been developed in the past for use in improving decisions and policies. Here we discuss the major types of models that produce response outputs that are of interest to decision/policy makers. First, statistical models have been developed using historical data sets on system responses, such as crop yield, milk production, and prices of commodities. For example, statistical models — fitting a function to predict crop yield using observed weather variables and crop regional yield statistics over multiple years — were the first crop models used for large-scale yield estimations. Average regional yields were regressed on weather and time to reveal a general trend in crop yields (Thompson, 1969). It is assumed that the data used to create statistical models are samples of a population such that the model can be used to predict regional yields in new years with different weather patterns.

In most cases, results of statistical models cannot be extrapolated “out of sample” because data used for parameter estimation do not represent the soil, management, weather and other conditions encountered elsewhere. Furthermore, they are poorly suited to estimate climate change impacts in the future because they cannot represent unobserved changes in management (adaptation), soil properties, pests and diseases, and the influence of increasing atmospheric CO_2_ concentrations (beyond the range of historical data). Despite these limitations, statistical models can be useful. When sufficient data are available to develop such models, they can provide insights about historical influences on past yields and inform other kinds of models (Antle, 1983, Lobell et al., 2011, Schlenker. et al., 2013, Tack et al., 2015). They also can be coupled with process-based models to predict out-of-sample responses (Antle and Capalbo, 2001).

A widely used approach for modeling agricultural systems can be classified as dynamic system simulation models. In contrast to the statistical approach, these models have functions that describe the changes in systems states in response to external drivers (e.g., weather and management practices), and how those changes are affected by other components in the system (see Wallach et al., 2014). This approach is used for all types of models, including crop, livestock, and farming system models, with model outputs being the values of model state variables over time (e.g., typically daily outputs for crop and pasture models). These dynamic models can be used to simulate multiple responses for the specific time and variables as needed (Wallach et al., 2014), and thus can compare effects of alternative decisions or policies on tradeoffs among those various responses. These dynamic system models may have mechanistic and functional components. Examples of dynamic models for cropping systems are those in the DSSAT suite of models (Jones et al., 2003), and APSIM (Keating et al., 2003), CROPSYST (Stöckle et al., 2003), and EPIC (Williams et al., 1983, Williams et al., 1989). However, because some of these models are extremely complex, containing many descriptive variables and parameters and thus requiring many inputs and long run times, reduced form or summary models are sometimes derived from much more complex models for specific purposes (e.g., Jones et al., 1999, Chikowo et al., 2008, Dzotsi et al., 2013). This approach is particularly useful when one wants to integrate crop models, for example, into models of more comprehensive agricultural systems such as economic analyses at farm, national, or global scales.

Similarly, dynamic livestock models include Ruminant (Herrero et al., 2013, Herrero et al., 1996); LiveSim (Rufino et al., 2009); CNCPS (Ruiz et al., 2002), Grazplan (Freer and Donnelly, 1997), GLEAM (Gerber et al., 2014), among others, and farming system models include IMPACT-HHM (Herrero et al., 2007), Gamede (Vayssières et al., 2009); IAT (Lisson et al., 2010); APSFARM (Rodriguez et al., 2014), and FARMSIM (van Wijk et al., 2009). For a detailed review see van Wijk et al. (2014).

One other point to make about the use of models for decision-making is the type of decision being considered. To date, many models have been developed to help inform tactical decisions, such as when to apply a pesticide, when to irrigate, or when to sell livestock. However, the models that are most useful for those kinds of decisions are narrow in scope. They are not about how to best manage a crop for multiple inputs over a full growing system altogether, but simply when to perform those predetermined management operations. They only predict when a particular threshold is reached that has previously been shown to provide effective management. To address these broader decisions, a cropping system model might be used to develop apps in the future for use on smart phones or other hand held devices (e.g., see www.agroclimate.org; Fraisse et al., 2006, Janssen et al., 2017 — a paper in this special issue).

For planning and strategic decisions, multiple responses and tradeoffs are usually of interest to users. Dynamic models of component subsystems (e.g., simulating daily growth and partitioning of biomass) can be used to represent functional responses (e.g., end of season grain, biomass yield, or residues in response to a range of nitrogen fertilizer use). Ideally, such model-simulated responses can be used to infer responses by real systems. Virtual experiments (simulations) using the models can thus complement real experiments, but there is a need to evaluate model responses relative to real system responses for a range of conditions to establish confidence in the model and also provide a measure of uncertainty. Little has been done to establish uncertainty of agricultural systems models until recently (e.g., Rosenzweig et al., 2013a, Rosenzweig et al., 2013b, Asseng et al., 2013).

### 3.3 Spatial and temporal scales of agricultural system models

**Fig. 2:**
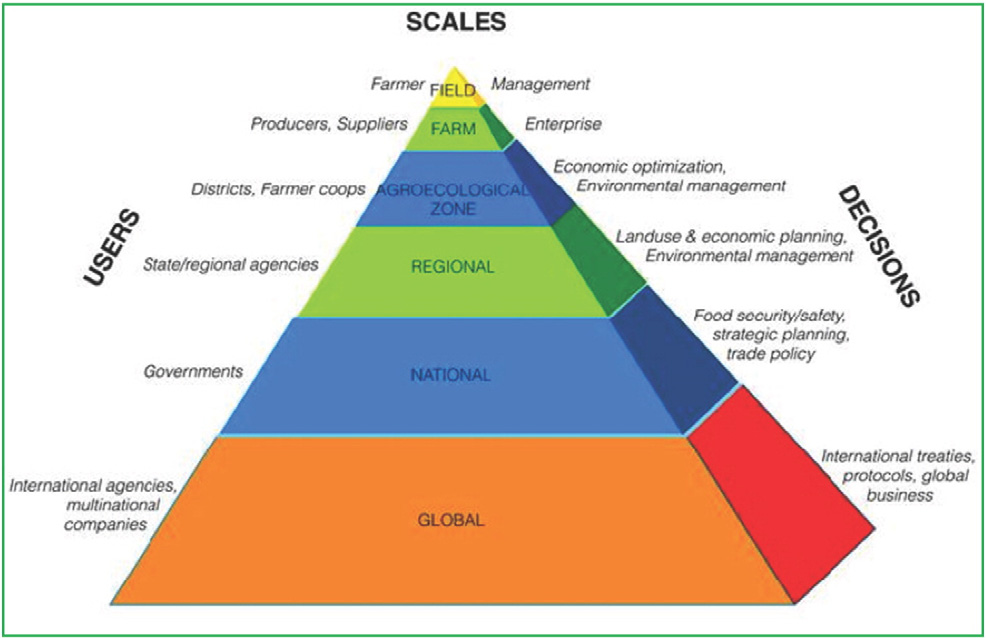
Scales/levels at which agricultural system models are developed along with types of users and decisions and policies of interest.

Users of models or information derived from them and the models themselves vary considerably across spatial and temporal scales as indicated in Fig. 2. Similarly, the scope of the system being modeled and managed varies depending on the questions being asked and the decisions and policies that are being studied. Users in Fig. 2 are not necessarily those who run the models; instead, they are those who want information about responses of the systems to different ways of managing them in whatever physical, biological, and socioeconomic climate conditions are involved. Thus, for example, a set of simulation experiments would be conducted by a researcher or advisor to address specific questions about alternative decisions or policies to help them make more informed decisions. Results from the simulation experiments could be summarized into advisory fact sheets or policy briefs for users. Or, results could be summarized in decision-support systems that are designed to provide information for key decisions of users (e.g., see www.agroclimate.org that targets extension agent and farmer users). Participatory modeling, where the development of a model is accomplished by model developers and stakeholders working together and discussing model results to refine simulations and better represent stakeholders' objectives, has also been successfully used (Thornton and Herrero, 2001, McCown, 2002).

#### 3.3.1 Field level

The scope of the system is important in determining what type of model is needed and what users are being targeted. Agricultural system models have been developed at all of the hierarchical levels shown in Fig. 2. The system could be at the field level where one wants to know the best management practices that meet production, profitability, and environmental protection goals of a farmer who is producing the crop in an area with strict environmental regulations (e.g., He et al., 2012). Cropping system models are used to predict how much economic yield the crop will produce and how much nutrient leaching would occur under different combinations of management practices and crop seasons. Similarly, the system could be a livestock-system managed by a rancher or dairyman. Livestock models are designed to predict herd or animal performance under different combinations of breeds and management. They may predict the number of livestock of different ages by sex and the body mass, or milk production per day of each lactating cow, all influenced by herd management and marketing of meat, calves, and/or milk. Thus, at the field or enterprise level, biophysical models are used to analyze responses similar to the way that experiments on the real systems would be analyzed. In many cases, these models are used to perform simulation experiments in combination with limited treatments in real experiments to help provide confidence in simulated experiment results when extending them to a wider range of options than could be tested in the real world.

Field level models usually assume homogeneity of conditions horizontally across the field, but may consider that the soil properties vary vertically with depth. Spatially homogeneous models are also referred to as “point” models implying that all points in a field area have the same properties. Some modern models support precision agriculture research and practice by incorporating heterogeneity of field conditions (e.g., Sadler and Russell, 1997, Paz et al., 2001, Basso et al., 2001, Basso et al., 2013, Braga et al., 1999). For example, point or field models (e.g., crop models) are also used to simulate responses at more aggregate scales by providing them with spatially-varying inputs (e.g., spatially-variable vertical soil properties, daily weather data, and/or management). For example, a crop model may be used to simulate multiple homogeneous fields across a farm, each with its own set of input conditions. As such, models of any particular level in Fig. 2 may be represented by multiple instances of models of smaller areas, thereby serving as building blocks in a hierarchical sense. Powerful computer systems allow field-level models to be scaled to regional or even national levels, if suitable input data are available (Elliott et al., 2014a, Elliott et al., 2014b).

#### 3.3.2 Farm and broader levels

An agricultural system could also be defined as a farm with land area on which different crops and livestock are produced, each of which is managed by a farm family or business entity. In this case, the enterprises of a farm interact in various ways, as described later. At a broader spatial scale, one may define an agricultural system as the land area in a region, district, or landscape that produces a particular commodity or various crops. The system model for that set of users predicts total production of the crop or crops in that area as affected by weather, soil, management and socioeconomic conditions, including a capability to evaluate decision and policy options. This landscape or regional model may also predict the amount of nutrient leaching or soil erosion for particular practices and policies being analyzed. Depending on the goals of the users, different approaches are used to develop the system model. But, typically at this scale, the models should include biophysical responses of crops and livestock as well as socioeconomic, environmental, policy, and business issues. These same characteristics of system models are important at national and global scales, in that biophysical, socioeconomic, and policy components are needed to model the important interactions and production, environmental, and economic responses to different decision and policy options. Recent years have seen increased interest in studying the interaction of agro-ecosystems with other managed and unmanaged ecosystems. This has several motivations, including understanding the importance of ecosystems services such as pollination and biological pest control provided to agriculture by natural habitats as well as issues of managing biodiversity in landscape mosaics (or more generally managing multifunctional landscapes). Another major application has been the analysis of potential for agricultural greenhouse gas mitigation through soil carbon sequestration.

Fig. 2 concerns users that range from farmers to policy makers and businesses that are interested in improving decisions and policies ranging from field, landscape, regional, national, and global scales. The delineation of the land area over which decisions and policies are made varies considerably, depending on the stakeholder/user and his/her interests. At each scale, the landscape can be decomposed into areas delineated by agro-ecological boundaries (such as a watershed) or into areas delineated by socioeconomic boundaries, such as the political boundaries of a district or country. Models at each of these scales may be developed by using component models of smaller areas. For example, a national model may make use of field scale crop models to simulate production across many districts then aggregated to the national scale for use in an economic model of the policy impacts on the aggregate production or its variations across districts. An alternative to this approach would be to use an aggregate national production model.

Agricultural system models at each of the scales in Fig. 2 are imperfect predictors of real system performance. To quote two famous statisticians, “All models are wrong, but some are useful” (Box and Draper, 1987, p. 424). Model developers make assumptions about what components to include in the system, how these components interact, and how they respond to the environment and to management practices and policies. The models themselves and their performances also depend heavily on the data used to develop and evaluate them. As a result, there is considerable uncertainty in results produced by the use of agricultural systems models. One of the important areas for improvement in the next generation of data, models and knowledge products linked to them is a better understanding, characterization and communication of this uncertainty to model users (Antle et al., 2017a--in this issue).

## 4 Discussion

The history of agricultural systems modeling shows that major contributions have been made by different disciplines, addressing different production systems from field to farm, landscape, and beyond. In addition, there are excellent examples in which component models from different disciplines have been combined in different ways to produce more comprehensive system models that consider biophysical, socioeconomic, and environmental responses. There are many examples where crop, livestock, and economic models have been combined to study farming systems as well as to analyze national and global impacts of climate change, policies, or alternative technologies, as shown in the companion paper on the state of agricultural system science (Jones et al., 2017--in this issue). This history also shows that the development of agricultural system models is still evolving through efforts of an increasing number of research organizations worldwide and through various global efforts, demonstrating that researchers in these institutions are increasingly interested in contributing to communities of science (e.g., via the global AgMIP, 2014 effort (www.agmip.org), various CGIAR-led programs, e.g., such as the IFPRI-led Global Futures and Harvest Choice projects (www.ifpri.org/) and the CIAT-led CCAFS project (ccafs.cgiar.org/)), the new CIMSANS Center www.ilsi.org/ResearchFoundation/CIMSANS/Pages/HomePage.aspx, and various global initiatives that aim for more harmonized and open databases for agriculture.

This history demonstrates that a minimum set of component models are needed to develop agricultural system models that are more or less common across various applications. These include crop models that combine weather, soil, genetic, and management components to simulate yield, resource use, and outputs of nutrients and chemicals to surrounding water, air, and ecological systems. These crop models need to take into account weed, pest and disease pressures, and predict performance to a range of inputs and practices that represent subsistence to highly controlled, intensive production technologies and new varieties. Similarly, livestock models are needed that account for climate, herd management, feed sources, and breeds. Farming system models are needed that integrate the various livestock and cropping systems, including their interactions, taking into account the socioeconomic and landscape characteristics of specific farms and a population of farms to address questions by individual farmers, agribusiness, and policy makers at community to subnational, national, and global scales. Similarly, this commonality should provide incentive for the efforts at creating harmonized and open databases to ensure that these basic needs for data will address future needs. The history also led us to conclude that different platforms for combining models and data for specific purposes will be necessary, and that the design of next generation models and data should account for this need over a range of platforms for applying the models and providing outputs needed for the various use cases that exist, as illustrated by those presented in the introduction to this special issue (Antle et al., 2017a--in this issue).

Several key lessons and important messages emerge from this history. These lessons should be considered by those who want to create an enabling environment for development of next generation agricultural system models and to help the community of developers avoid roadblocks and pitfalls. Here we summarize these key lessons.

• *Capitalize on crises*. This history shows that major advances in agricultural systems modeling occurred when there were food security concerns or other crises and then decelerated afterward. Other studies (e.g., Royal Society, 2014) concluded that often policy shifts occur only after a major disaster. Thus, it is important that we have the science and analytical tools available beforehand to act quickly to get things done while there is a window of opportunity.
• *Technological advances*. A strong lesson from the past is the influence of technological advances, including mainframe computers, the PC, and the Internet. New technologies and knowledge should be embraced by those who are developing next generation of agricultural systems models, data, and knowledge systems. Contemporary technology examples include smart phones and telecommunications, apps and video games, molecular biology, remote sensing, open source software tools, cloud computing as a means of enabling broad access to powerful tools (Foster, 2011, Montella et al., 2015), and high-performance parallel computers for large parameter sweeps, model comparisons, and gridded crop model simulations (Elliott et al., 2014a, Elliott et al., 2014b).
• *Open, harmonized data*. Most agricultural system models have been developed using relatively narrow ranges of data, mainly because most modelers have collected their own data sets in order to develop a model. Although there are exceptions, data obtained by most biophysical agricultural scientists are lost soon after researchers collect the data and publish their results. Metadata, standards and protocols are needed to harmonize the databases that exist and to facilitate entry of data that are now mostly lost after collection and primary use. International collaborations such as the Global Open Data for Agriculture and Nutrition initiative (GODAN, www.godan.info) are encouraging the opening up of agricultural datasets, and research funders are bringing in open data policies for the research that they fund.
• *Transdisciplinarity*. Major advances have occurred in the past when different disciplines joined forces, such as occurred when crop modeling and remote sensing scientists collaborated to create models for predicting wheat yield worldwide in the 1970s, or when crop models joined up with land-surface scheme models to start to simulate crops within numerical climate models in the 2000s. There is a need to broaden the collaboration, such as now exists to some degree among biophysical and economic modelers, in particular to include plant and animal breeders, insect and disease researchers and modelers, etc. Transdisciplinary collaboration between the more fundamental ecologists/epidemiologists and those involved directly in practical pest and disease management is needed.
• *Modularity and interoperability*. The agricultural systems research community needs to have standards and protocols so that they can access and use the same sources of data “in the cloud” from multiple sources and to operate multiple models, knowledge products, and decision support systems. It is important to have different models and approaches, but we need to develop standards and protocols to fully gain the benefits from these developments. We now know that it does not pay to aim for only one “perfect” model. Instead, we should aim for component models that are structured as modules that can be used alone to address specific questions (such as when to apply a chemical or irrigation) and, more importantly, where those modules can be integrated into holistic biophysical and economic models to address more comprehensive problems. Modular models are needed to ensure efficient scientific progress as well as model longevity and maintainability.
• *User-driven data and model development*. The history of data and model development shows that many existing models have been developed for research purposes and then adapted to address user needs. Most models remain “user unfriendly” and while some models are linked to DSS software, it remains difficult for many users to access model outputs or to otherwise make use of models. With the rapid advances in information and communications technology, it is now clear that there is a large unrealized potential for data and models to be more effectively utilized through various kinds of “knowledge products” including computer visualization tools and mobile technology.

Through the review of existing initiatives and discussions among the authors involved in this special issue, it is clear that there is a need for a more focused effort to connect these various agricultural systems modeling, database, harmonization and open-access data, and DSS efforts together, so that the scientific resources being invested in these different initiatives will contribute to compatible set of models, data, and platforms to ensure global public goods. This is critically important, considering that these tools are increasingly needed to ensure that agriculture will meet the food demands of the next 50 to 100 years and will be sustainable environmentally and economically.
